# Hyaluronic Acid/Chondroitin Sulfate-Based Dynamic Thiol–Aldehyde Addition Hydrogel: An Injectable, Self-Healing, On-Demand Dissolution Wound Dressing

**DOI:** 10.3390/ma17123003

**Published:** 2024-06-19

**Authors:** Melissa Johnson, Rijian Song, Yinghao Li, Cameron Milne, Jing Lyu, Irene Lara-Sáez, Sigen A, Wenxin Wang

**Affiliations:** 1 Charles Institute of Dermatology, School of Medicine, University College Dublin, D04 V1W8 Dublin, Irelandjing.lyu@ucd.ie (J.L.); irene.lara-saez@ucd.ie (I.L.-S.);; 2 School of Medicine, Anhui University of Science and Technology, Huainan 232001, China; 3 Research and Clinical Translation Center of Gene Medicine and Tissue Engineering, School of Public Health, Anhui University of Science and Technology, Huainan 232001, China

**Keywords:** dynamic covalent chemistry, thiol–aldehyde addition, wound healing, self-healing, on-demand dissolution

## Abstract

Frequent removal and reapplication of wound dressings can cause mechanical disruption to the healing process and significant physical discomfort for patients. In response to this challenge, a dynamic covalent hydrogel has been developed to advance wound care strategies. This system comprises aldehyde functionalized chondroitin sulfate (CS-CHO) and thiolated hyaluronic acid (HA-SH), with the distinct ability to form in situ via thiol–aldehyde addition and dissolve on-demand via the thiol–hemithioacetal exchange reaction. Although rarely reported, the dynamic covalent reaction of thiol–aldehyde addition holds great promise for the preparation of dynamic hydrogels due to its rapid reaction kinetics and easy reversible dissociation. The thiol–aldehyde addition chemistry provides the hydrogel system with highly desirable characteristics of rapid gelation (within seconds), self-healing, and on-demand dissolution (within 30 min). The mechanical and dissolution properties of the hydrogel can be easily tuned by utilizing CS-CHO materials of different aldehyde functional group contents. The chemical structure, rheology, self-healing, swelling profile, degradation rate, and cell biocompatibility of the hydrogels are characterized. The hydrogel possesses excellent biocompatibility and proves to be significant in promoting cell proliferation in vitro when compared to a commercial hydrogel (HyStem^®^ Cell Culture Scaffold Kit). This study introduces the simple fabrication of a new dynamic hydrogel system that can serve as an ideal platform for biomedical applications, particularly in wound care treatments as an on-demand dissolvable wound dressing.

## 1. Introduction

Wound healing is a complex dynamic process characterized by four interconnected phases: hemostasis, inflammation, proliferation, and remodeling [1,2]. When factors inhibit the wound healing process, a chronic wound can develop, posing a tremendous burden on both the healthcare system and the patient [3]. Today, the clinical demand for advanced wound dressings is consistently increasing with the prevalence of chronic diseases and surgical procedures [4]. As a result, different types of wound dressings have been explored, such as gauze [5], films [6], foam dressings [7], hydrocolloid dressings [8,9], wound fillers [10], and hydrogel dressings [11,12]. 

In recent years, hydrogels have constituted one of the most investigated materials for wound healing applications [13]. Hydrogels are three-dimensional networks of hydrophilic polymers that can be derived from both natural polymers (e.g., hyaluronic acid, chondroitin sulfate, chitosan, alginate, and collagen) and/or synthetic polymers (e.g., poly (ethylene glycol) (PEG), poly (acrylic acid) (PAA), and poly (vinyl alcohol) (PVA)) [14,15,16]. In particular, hydrogels based on natural-derived polymers have gained increasing attention due to their inherent biodegradability and biocompatibility [17]. 

To extend the range of clinical hydrogel wound dressings, dynamic covalent crosslinking hydrogels have emerged as the most promising wound dressing materials [18]. Dynamic covalent hydrogels contain dynamic covalent bonds that can be broken and reversibly re-formed under certain stimuli, which endows dynamic hydrogels with excellent properties such as self-healing and injectability [19]. The most frequently explored dynamic covalent interactions for biomedical applications include imine and hydrazone (e.g., for pH-responsive drug delivery), disulfide and thioester exchange (e.g., for degradation-controlled wound dressings), boronic ester formation (e.g., for glucose-responsive drug delivery), and Diels–Alder (e.g., for drug delivery and cellular scaffolds) [18,20,21,22].

In situ-formed injectable hydrogels can form under physiological conditions which allows irregular and/or deep wounds to be covered. Subsequently, it is important that wound dressings that adhere to the wound surface are replaced on a regular basis once they have served their purpose. In clinical practice, dressing changes necessitate cutting and mechanical debridement, resulting in tissue damage, delayed healing, and personal suffering for the injured patient. As a result, hydrogel dressings that can dissolve on-demand have a high potential in the clinic as they can decrease procedurally induced tissue damage and physical pain. The thiol–thioester exchange reaction, thiol–disulfide exchange reaction, retro Diels–Alder reaction, and retro Michael-type addition are the most commonly used dynamic chemical crosslinking (DCC) techniques to prepare on-demand dissolution hydrogels [23,24]. 

Typical retro-Michael reaction-based hydrogels utilize maleimide (MAL)-functionalized macromolecular monomers crosslinked with thiol-functionalized multi-arm polymers to form thioether crosslinked networks [24]. A. Baldwin et al. formed in situ dissolvable hydrogels by mixing thiolated 4-arm-PEG and MAL-functionalized heparin which were subsequently dissolved by the addition of glutathione [25]. However, the slow dissolution rate of high-stability DCC hydrogels limits their application as dissolvable wound dressings [23]. To overcome this limitation and meet sophisticated clinical requirements, it is critical to develop a variety of dynamic chemistry methods, and the study of thiol–aldehyde addition, though rarely reported, holds great promise for the preparation of on-demand dissolution dynamic hydrogels due to its rapid reaction kinetics and easy reversible dissociation via the thiol–hemithioacetal exchange reaction [26,27].

Y. Hua et al. developed a moldable and removable hydrogel wound dressing. The hydrogel system was fabricated by dynamic crosslinking of thiolated PEG polymers with oxidized dextran through hemithioacetal formation [28]. The labile hemithioacetal bonds easily exchanged with a thiol source, GSH, leading to wound dressing removal ability, preventing secondary injuries to the skin. However, the dynamic hydrogels had a slower gelation period ranging from 700 to 3600 s. Similarly, R. Yang et al. developed a range of hydrogels fabricated by dynamically crosslinking thiolated poly (γ-glutamic acid) and oxidized hyaluronic acid [29]. A shorter gelation time (10 s) was observed at high solid polymer concentrations, however, all hydrogel samples degraded within 1 h in PBS solution (pH 7.4).

**Scheme 1:**
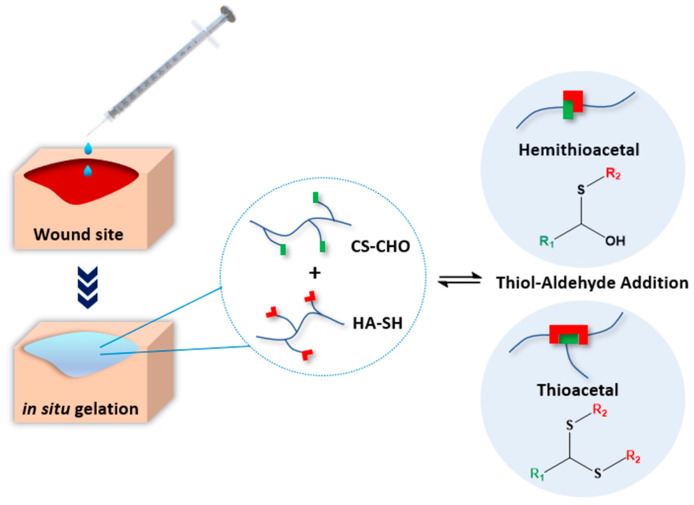
A schematic illustration of the concept of injectable CS-CHO/HA-SH hydrogel formation. The hydrogel is in situ formed via thiol–aldehyde addition by mixing CS-CHO and HA-SH solutions.

Therefore, we hypothesize that increasing the functional group content within a dynamic hydrogel system, utilizing naturally derived functionalized polysaccharides, could reduce gelation time while enhancing mechanical properties and biocompatibility. As a result, in this study, a dynamic covalent hydrogel based on thiol–aldehyde addition was designed to address the major unmet need for a wound dressing that (1) forms in situ with fast gelation, (2) is self-healing, (3) can be easily and atraumatically removed under controlled conditions, (4) is biocompatible, and (5) can combine with active substances that can promote wound healing (e.g., stem cells, drugs, growth factors, and exosomes). The hydrogels were prepared by mixing an aldehyde-functionalized chondroitin sulfate (CS-CHO) solution and a thiolated hyaluronic acid (HA-SH) solution (Scheme 1). To improve the hydrogel’s mechanical properties, CS-CHO with a higher aldehyde functional group content was used to increase the crosslinking density in the dynamic network. The chemical structure, rheology, swelling profile, degradation, and cell biocompatibility of the hydrogels were characterized, and the findings demonstrate that the hydrogel exhibits fast gelation, self-healing properties, injectability, on-demand dissolution capability, and excellent biocompatibility. These attributes position the hydrogel as a promising candidate for wound care applications, particularly as a dissolvable wound dressing. Therefore, the combination of dynamic thiol–aldehyde addition chemistry with functionalized polysaccharides offers a versatile and effective solution to address critical unmet needs in wound care.

## 2. Materials and Methods

### 2.1. Materials

Chondroitin sulfate C sodium salt (sulfur content: 5.1%, laboratory research grade) was purchased from Biosynth (Gardner, MA, USA). Hyaluronic acid (220 kDa, food grade) was purchased from Stanford chemical Ltd. (Lake Forest, CA, USA). Sodium (meta) periodate (99.5%), 1-Ethyl-3-[3-(dimethylamino)propyl]carbodiimide (EDCI), ethylene glycol (99.8%), dithiothreitol, sodium chloride, hydrochloric acid (34%), sodium hydroxide (≥98%), 5,5-dithio-bis-(2-nitrobenzoic acid) (Ellman’s Reagent), L-cysteine hydrochloride, ethylenediaminetetraacetic acid (≥98.5%), t-butyl carbazate (98%), 2,4,6-trinitrobenzenesulfonic acid solution (TNBS, 5% (*w*/*v*) in H_2_O), sodium tetraborate decahydrate (≥99.5%), trichloroacetic acid (98%), phosphate buffered saline, and glutamic acid L-cysteine were purchased from Sigma-Aldrich (St. Louis, MO, USA) and were used without purification. The 3,3′-dithiobis(propanoic dihydrazide) (DTP) was synthesized according to the method described in the ‘Supplementary Experimental Section’. Dialysis tubing (cut off Mw 8 kDa) was purchased from Spectrum Lab (San Francisco, CA, USA). Normal Human Dermal Fibroblasts (NHDFs) were purchased from ATCC (Manassas, VA, USA). Dulbecco’s modified Eagle’s medium (DMEM), fetal bovine serum (FBS), and penicillin/streptomycin were acquired from Invitrogen (Waltham, MA, USA). AlamarBlue agent, a Live/Dead Viability Cytotoxicity Kit, and a HyStem^®^ Cell Culture Scaffold Kit were purchased from Sigma-Aldrich.

### 2.2. Instruments

Rheological assessments were performed using a TA HR-2 rheometer (TA Instruments, New Castle, DE, USA) fitted with an 8 mm steel parallel plate geometry. A microplate reader (Molecular Devices SpectraMax M3, San Jose, CA, USA) was used to collect the absorbance data. A Leica microscope (DM2500 fluorescence, Leica Microsystems, Wetzlar, Germany) was utilized to detect cell staining.

### 2.3. Methods

#### 2.3.1. Synthesis of Aldehyde-Functionalized Chondroitin Sulfate (CS-CHO)

CS-CHO was synthesized according to a previously published procedure with modifications to produce different degrees of oxidation [30]. Chondroitin sulfate (CS) (1.19 g, 11.88 g) was dissolved in deionized H_2_O (100 mL) and then sodium periodate (1 equiv.) was added slowly. The reaction solution was stirred at RT in the dark for 4 h. Then, ethylene glycol (0.5 mL and 5 mL, respectively) was added to consume the excess periodate. The products were named CS-CHO(L) and CS-CHO(H), respectively. The letters ‘L’ and ‘H’ indicate the lower and higher oxidation degrees of CS-CHO. The solution was stirred for 1 h. For purification, the reaction solution mixture was transferred to dialysis tubing with a molecular weight cut-off of 8 kDa and dialyzed against deionized H_2_O for 4 days, with 12 water changes during this period. Subsequently, the dialyzed solution was lyophilized to yield a white solid foam.

#### 2.3.2. Characterization of CS-CHO by 2,4,6-Trinitrobenzene Sulfonic Acid (TNBS) Assay

A total of 30 µL (0.6%) CS-CHO and 30 µL (30 mM) t-butyl carbazate (t-BC) in an aqueous solution of trichloroacetic acid (1%) were mixed and left to react at room temperature. After 24 h, a 0.6 mL TNBS solution (6 mmol, 0.1 M borate buffer, pH 8.0) was added to the solution mixture to react with the t-BC. The reaction solution was left to react for 1 h at room temperature. A volume of 200 µL of the mixture was diluted with 400 µL of 0.5 N HCl and allowed to react for 30 min. A volume of 150 µL of the final mixture was transferred into a 96-well plate. The absorbance of the solution was measured with a spectrophotometer microplate reader at 340 nm. A standard calibration curve derived from the aqueous t-BC solutions (5–30 mM) was used to determine the quantity of unreacted t-BC and calculate the aldehyde content result. All experiments were completed in triplicate.

#### 2.3.3. Synthesis of Thiolated Hyaluronic Acid (HA-SH)

HA-SH was synthesized according to a previously published procedure with minor modifications [31]. Hyaluronic acid (HA) ((220 kDa), 2 g) was dissolved in deionized H_2_O (200 mL) and then 3,3′-dithiobis(propanoic dihydrazide) (DTP) (3.5740 g, 7.5 mmol) was added with continuous stirring of the solution. The pH of the reaction solution was adjusted to 4.8 through the addition of 1.0 M of HCl. Next, EDCI (2.8756 g, 7.5 mmol) was added. The pH of the reaction mixture was kept at 4.75 by adding aliquots of 1.0 M of HCl. The reaction was stopped by adding 1.0 M of NaOH to raise the pH of the reaction solution to 7.0. Subsequently, DTT (10.0264 g, 32.50 mmol) was added, and the pH of the solution was adjusted to 8.5 by adding 1.0 M of NaOH. After the solution was stirred for 24 h, it was transferred to dialysis tubing with a molecular weight cut-off of 8 kDa. It was then dialyzed against dilute HCl (pH 3.5) containing NaCl (100 mmol), followed by dialysis against dilute HCl (pH 3.5). Subsequently, the purified solution was lyophilized to yield a white solid foam, which was stored under an argon atmosphere. 

#### 2.3.4. Characterization of HA-SH by Ellman’s Assay

The HA-SH sample solution (1 mg/mL) was prepared in a reaction buffer (0.1 M sodium phosphate, 1 mM EDTA, pH 8.0) and a standard curve of L-cysteine hydrochloride (0–30 mM) was prepared and mixed with Ellman solution (4 mg/mL) for 30 min. The absorbance was measured at 412 nm using a microplate reader. The concentration of the sample solution was determined by comparison with the standard curve of L-cysteine hydrochloride.

#### 2.3.5. Characterization of HA-SH by ^1^H-NMR Spectroscopy

The ^1^H-NMR data for HA-SH were obtained using a Varian 400 MHz spectrometer (Varian, Palo Alto, CA, USA), and D_2_O was used as the deuterated solvent. Tetramethylsilane (TMS) at 0 ppm was utilized as the internal standard at a concentration of 0.03% (*v*/*v*). Chemical shifts were reported in parts per million (ppm).

#### 2.3.6. Hydrogel Fabrication of CS-CHO/HA-SH

The CS-CHO solutions were prepared by dissolving the synthesized CS-CHO in PBS (4% *w*/*v*). The HA-SH solutions were prepared by dissolving the synthesized HA-SH in PBS (4% *w*/*v*). The hydrogel was formed by mixing the CS-CHO solution and HA-SH solution at a 1:1 volume ratio at RT.

#### 2.3.7. Visual Hydrogel Self-Healing Test 

The spontaneous self-healing behavior was visually determined. A ~2.5 cm diameter round hydrogel disk was methylene blue-stained to aid visual examination. A hole of an approximately 1.0 cm diameter was punctured at the center of the hydrogel. Photos were then captured at different time intervals to observe and visualize the self-healing process.

#### 2.3.8. Hydrogel Rheological Assessments

A TA rheometer (HR-2) fitted with an 8 mm parallel plate was used to measure the mechanical properties of the hydrogels prepared. The premixed solution was pipetted between the parallel plates for time sweep tests, and the tests were performed immediately at 25 °C with a frequency of 1.0 Hz and a strain of 1% for 1800 s. A strain sweep test was conducted at 25 °C with a frequency of 1.0 Hz from 1% to 1000% strain to evaluate the linear viscoelastic properties. Self-healing properties were tested at 25 °C through a continuous oscillatory strain between 1% and 200% (CS-CHO(H)/HA-SH) and between 1% and 100% (CS-CHO(L)/HA-SH) at 1.0 Hz with 60 s for each step. The strain sweep and self-healing tests were conducted using cylinder hydrogel samples (200 µL) and the testing gap for each test was 1000 µm. Each test was carried out in triplicate. The data collected were analyzed by TRIOS software (version 5.1).

#### 2.3.9. In Vitro Hydrogel Swelling and Degradation Study

Swelling and degradation studies were conducted on CS-CHO(H)/HA-SH and CS-CHO(L)/HA-SH hydrogels. The swelling/degradation profile was determined by adding 2 mL of PBS solution (pH 7.4) to the glass vials to immerse the hydrogels. The glass vials were placed into a shaker at 37 °C and 150 rpm. At each scheduled time point, the hydrogels were extracted, gently blotted with filter paper to remove the surface solution, and then weighed (W_t_). Each sample was conducted in triplicate. The swelling profile and degradation rate were calculated from the following equation:

Percentage of hydrogel mass (%) = W_t_/W_0_ × 100%

W_t_ represents the weight at the scheduled time point, and W_0_ denotes the initial weight of the hydrogel.

#### 2.3.10. Hydrogel Dissolution Behavior Study

Hydrogel dissolution behavior studies were conducted on CS-CHO(H)/HA-SH and CS-CHO(L)/HA-SH hydrogels. A total of 2 mL of PBS solution with different concentrations of cysteine (0.01 M, 0.05 M, 0.10 M, and 0.30 M) or glutamic acid (0.1 M) was added to the glass vials to immerse the hydrogels (the pH of all dissolution solutions was adjusted to pH 7.4). At each scheduled time point, the hydrogels were extracted, gently blotted with filter paper to remove the surface solution, and then weighed (W_t_). Each sample was conducted in triplicate. The dissolution behavior was calculated from the following equation:

Percentage of hydrogel mass (%) = W_t_/W_0_ × 100%

W_t_ represents the weight at the scheduled time point, and W_0_ denotes the initial weight of the hydrogel.

#### 2.3.11. Cell Culture

Human keratinocyte (HaCaT) cells and Normal Human Dermal Fibroblasts (NHDFs) were cultured in full cell media, Dulbecco’s Modified Eagle Medium (DMEM) with 10% fetal bovine serum (FBS) and 1% penicillin under standard cell culture conditions (37 °C, 5% CO_2_). The HaCaTs were seeded in a 96-well plate at a density of 1 × 10^4^ cells/well for the cytotoxicity test of the functionalized polymer solutions. The NHDFs were seeded in a 24-well plate at the density of 1 × 10^5^ cells/well for the cell viability of CS-CHO(H)/HA-SH hydrogels. 

#### 2.3.12. Cytotoxicity Test of CS-CHO and HA-SH Polymer Solutions

The cytotoxicity assessment of CS-CHO and HA-SH polymer solutions was tested using HaCaT cells by an alamarBlue assay. All solutions for cell viability tests were prepared using a PBS buffer and filtered for sterilization using a 0.22 µm pore size filter. The positive control (100% viability) was defined as cells without treatment. The HaCaTs were seeded in the 96-well plate. Following overnight culture, the cell media were replaced with a series of CS-CHO solutions (from 100 to 1000 µg/mL in full cell media, *n* = 3) or HA-SH solutions (from 100 to 1000 µg/mL in full cell media, *n* = 3). The cell viability was tested at 24 h and 72 h after co-culture using alamarBlue. After 4 h of incubation, the absorbance was measured at 570 nm with a microplate reader. Live/Dead kit (calcein/ethidium) staining was utilized to confirm the living status of the cells. A staining solution was prepared by adding 2 μL of calcein AM and 8 μL of ethidium homodimer-1 into DPBS. After 24 h and 72 h, the culture medium was removed and replaced with 100 μL of the Live/Dead stain. After 30 min of incubation at 25 °C, the stain was washed away three times from the well plates with PBS. The images were captured using a fluorescence microscope.

#### 2.3.13. Cell Viability of CS-CHO(H)/HA-SH Hydrogel

The cell viability assessment of the CS-CHO(H)/HA-SH hydrogel system was tested by seeding cells on the hydrogel surface. Briefly, CS-CHO(H) (4% *w*/*v*) and HA-SH (4% *w*/*v*) were fully dissolved in fresh medium and then filtered by a 0.22 μm filter. A total of 250 μL of the CS-CHO(H)/HA-SH hydrogel and a HyStem^®^ Cell Culture Scaffold Kit (fabricated according to the manufacturer’s protocol) were fabricated in the 24-well cell culture plates. After one hour, the NHDFs were collected by centrifugation before being seeded onto the surface of the hydrogels. The positive control was defined as cells seeded directly onto the cell plate without hydrogel. The cell viability was tested at 24 h and 72 h after co-culture using alamarBlue. After 4 h of incubation, the absorbance was measured at 570 nm with a microplate reader. Live/Dead kit (calcein/ethidium) staining was utilized to confirm the living status of the seeded cells. A staining solution was prepared by adding 2 μL of calcein AM and 8 μL of ethidium homodimer-1 into DPBS. After 24 h and 72 h, the culture medium was removed and replaced with 500 μL of the Live/Dead stain. After 30 min of incubation at 25 °C, the stain was washed away three times from the well plates with PBS. The images were captured using a fluorescence microscope.

#### 2.3.14. Statistical Analysis

The obtained values (*n* > 3) are expressed as the mean ± standard deviation (SD). For the CS-CHO(H)/HA-SH cell viability analysis, statistical differences between the two groups were determined using a Student’s unpaired *t* test. A value of * *p* < 0.05, ** *p* < 0.01, or *** *p* < 0.001 was considered statistically significant.

## 3. Results and Discussion

### 3.1. Synthesis and Characterization of CS-CHO and HA-SH

**Scheme 2:**
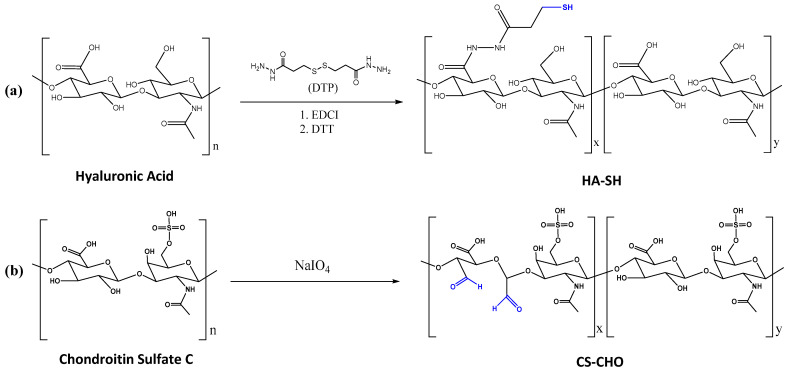
(**a**) Synthesis of thiolated hyaluronic acid (HA-SH); (**b**) synthesis of aldehyde functionalized chondroitin sulfate (CS-CHO).

Aldehyde functionalized chondroitin sulfate (CS-CHO) was obtained by the oxidation reaction of chondroitin-sulfate C (CS) (Scheme 2a) using sodium periodate (NaIO_4_), which oxidizes the adjacent hydroxyl groups (present at carbon 2 and 3 on the D-glucuronic acid unit) to form a periodate ester [30]. A cyclic mechanism then cleaved the C-C sigma bond, which led to the formation of two aldehyde groups.

By varying the concentration of the reactants, CS-CHOs of two different degrees of oxidation were synthesized: a higher oxidation degree (CS-CHO(H)) and a lower oxidation degree (CS-CHO(L)). The CS-CHO(H) product was prepared using a five-fold more concentrated reaction solution, while the molar ratio of CS to sodium periodate (1:1) remained constant in both reactions. The purified products were obtained as white foam after dialysis and freeze-drying, and the aldehyde content (mmol/g) of CS-CHO was determined by a TNBS assay. The resulting CS-CHO(L) and CS-CHO(H) aldehyde contents were 1.10 mmol/g and 1.87 mmol/g, respectively. The results of the aldehyde content show that as the reactant concentration increased, the rate of reaction increased, resulting in a higher aldehyde content.

HA-SH was obtained using EDCI chemistry (Scheme 2b) [31]. HA (1% *w*/*v*) was mixed with dithiobis(propanoic dihydrazide) (DTP), the pH was adjusted to 4.8, and then EDCI was introduced to initiate the reaction. The pH of the reaction solution was kept at 4.8 for two hours by the addition of HCl (1 M). The reaction was stopped by adding 1 M of NaOH to raise the pH of the solution to 7.0. The disulfide bonds were then reduced using DTT. The pH of the reaction solution was kept at 8.5 while stirring for 24 h at room temperature. The purified product was obtained as a white foam after dialysis and freeze-drying, and the free thiol content (mmol/g) of HA-SH was determined by Ellman’s assay. The free thiol content of the resulting HA-SH was 1.56 mmol/g. The HA-SH chemical structure was characterized by ^1^H-NMR. Figure S1 confirms the structure of the starting raw material of hyaluronic acid in comparison to HA-SH, which exhibits two new peaks at δ = 2.82–2.92 ppm and δ = 2.68–2.80 ppm. The degree of thiol functionalization was 84%, as determined by the comparative integration of the two new peaks relative to the peak at δ = 2.04 ppm. 

### 3.2. Fabrication of CS-CHO/HA-SH Hydrogels

CS-CHO(L)/HA-SH and CS-CHO(H)/HA-SH hydrogels were fabricated from CS-CHO(H) or CS-CHO(H) concentrations (4% *w*/*v*) and HA-SH concentrations (4% *w*/*v*), and the molar ratio of the thiol and aldehyde groups were calculated (Table 1). The hydrogels were obtained from a thiol–aldehyde addition reaction between the aldehyde groups of CS-CHO and the thiol groups of HA-SH. The resulting molar ratios suggest that the crosslinking network reaction occurs via hemithioacetal formation at an approximately 1:1 (-SH/-CHO) molar ratio, where one aldehyde group can react with at least one thiol group (Scheme 1). 

Both CS-CHO (4% *w*/*v*) and HA-SH (4% *w*/*v*) PBS solutions were flowable liquids, with a pre-gel solution pH of ~7.1. A stable hydrogel was formed within several seconds after mixing. The gelation time was monitored by using a magnetic stirrer bar to mix the CS-CHO solution in a glass vial. After the addition of the HA-SH solution, the viscosity of the solution immediately increased which prevented the magnetic stirrer bar from stirring. The gelation time for CS-CHO(L)/HA-SH was 9 s and decreased to 6 s for CS-CHO(H)/HA-SH, as the number of active aldehyde functional group sites was greater in CS-CHO(H) than CS-CHO(L) for crosslinking. The fast gelation time observed at a physiological pH enabled the hydrogel to meet the initial requirements for biomedical applications such as wound healing. 

### 3.3. Rheological Assessment of CS-CHO/HA-SH Hydrogels

**Figure 1:**
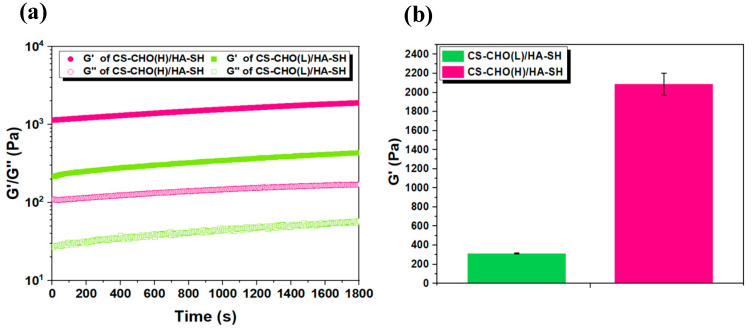
(**a**) Time sweep tests of the CS-CHO(H)/HA-SH and CS-CHO(L)/HA-SH hydrogels (25 °C, 1% strain, and 1 Hz); (**b**) storage modulus (G′) of CS-CHO(L)/HA-SH and CS-CHO(H)/HA-SH hydrogels at 24 h (25 °C, 1% strain, and 1 Hz).

To investigate the gelation behavior, a time sweep rheological test of the CS-CHO/HA-SH hydrogels was conducted. The storage modulus (G′) represents the hydrogel’s energy storage and elastic response, while the energy loss and viscous characteristics are represented by the loss modulus (G″). As shown in Figure 1a, the CS-CHO and HA-SH solution mixture immediately achieved a solid-like state when the storage modulus (G′) was greater than the loss modulus (G″). This immediate sol–gel transition was apparent for both of the hydrogels, due to the rapid kinetics of the thiol–aldehyde addition [32]. This immediate gelation characteristic is beneficial for fast application in wound management. Afterwards, the hydrogels had a stable network structure, as the G’ increased gradually and formed a stable plateau. The G′ and G″ of CS-CHO(H) were greater than CS-CHO(L)/HA-SH as with the increase in aldehyde content, the crosslinking density was improved. Overall, the G′ of CS-CHO(H)/HA-SH at 24 h was significantly higher than that of CS-CHO(L)/HA-SH, approximately 6.5 times greater, as shown in Figure 1b, due to the increased crosslinking density.

The strain amplitude sweep test (Figure S2), conducted 2 h after hydrogel formation, indicated the viscoelastic region of both hydrogel samples. The intersection of G′ and G″ at 32% and 158% occured for hydrogel samples CS-CHO(H)/HA-SH and CS-CHO(L)/HA-SH, respectively. Since CS-CHO(L)/HA-SH had a lower crosslinking density, it could undergo more strain before the G′/G″ crossover point, because the hydrogel network was less stiff compared to CS-CHO(H)/HA-SH, resulting in less deformation than higher crosslinked networks. The rheological results show that the CS-CHO/HA-SH hydrogels can be tailored to suit application demands. 

### 3.4. Self-Healing Property of CS-CHO/HA-SH Hydrogels

**Figure 2:**
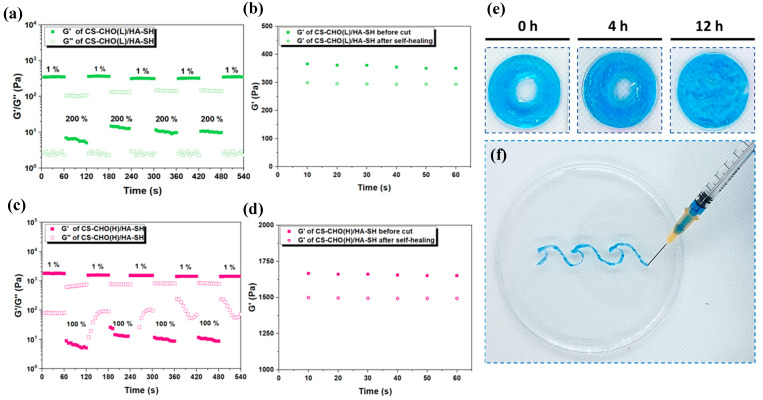
(**a**,**c**) Oscillation step strain test of applied strain from 1% to 200% and 1% to 100% for 540 s for CS-CHO(L)/HA-SH and CS-CHO(H)/HA-SH, respectively (25 °C, 1 Hz); (**b**,**d**) mechanical property test, before cut and after self-healing of CS-CHO(L)/HA-SH and CS-CHO(H)/HA-SH, respectively; (**e**) photographs of the self-healing process of CS-CHO(H)-HA-SH through the disappearance of the central hole in the hydrogel at room temperature; (**f**) CS-CHO(H)/HA-SH hydrogel was injected through a 28 gauge needle. The hydrogels were stained blue for observation.

The self-healing properties of the hydrogels were assessed through an oscillation step strain test to confirm the recovery of rheological properties following damage to the hydrogel network at a higher strain (Figure 2a,c). The test began at a 1% strain for 60 s, followed by a higher strain (200%, 100%) application for 60 s for CS-CHO(L)/HA-SH and CS-CHO(H)/HA-SH, respectively. Since CS-CHO(L)/HA-SH requires a higher strain before the G′/G″ crossover point, a higher strain was applied during the test. G′ decreased and became lower than G″ for both hydrogels as increased strain was applied. The G′ recovered fast when the strain was relieved and the recovery process was consistent for the 4 step strain cycles, indicating the excellent self-healing ability of the hydrogels due to its dynamic crosslinking network. To further assess the mechanical properties of the hydrogels before and after self-healing, the G′ was monitored after the hydrogel was cut in half and placed back together (Figure 2b,d). The small difference in G′ between before and after the cut suggested that the hydrogel network had been almost completely restored.

To visually demonstrate the self-healing ability of the dynamic hydrogel, CS-CHO(H)/HA-SH disks were fabricated and stained with methylene blue (Figure 2e). After the puncture of the hydrogel disk with a hole, the central hole diminished with time and finally disappeared after 12 h, demonstrating the excellent self-healing ability within the dynamic hydrogel system. In addition, the CS-CHO(H)/HA-SH hydrogel could easily be injected through a 28-gauge needle (Figure 2f). Overall, the excellent self-healing properties reduce the risk of wound infection caused by unintentional rupture of hydrogels, and the shear-thinning injectability property results in an equally covered wound that matches the practical operation criteria for an adaptable wound dressing.

### 3.5. In Vitro Swelling and Degradation Profile and On-Demand Dissolution Property

**Figure 3:**
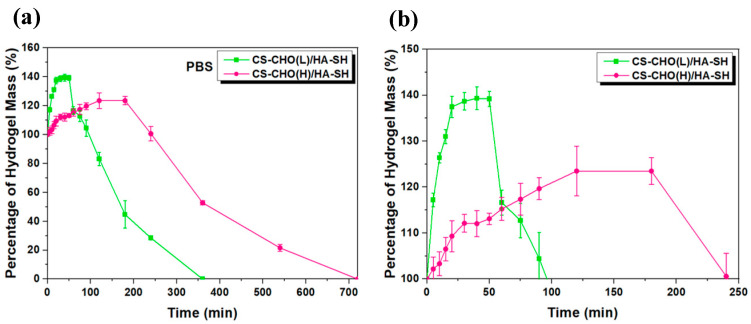
(**a**) Swelling and degradation profile of CS-CHO(L)/HA-SH and CS-CHO(H)/HA-SH (PBS, 37 °C); (**b**) magnification of (**a**) for the swelling profile section from 0 to 250 min.

The in vitro swelling property and degradation behavior of the CS-CHO(L)/HA-SH and CS-CHO(H)/HA-SH hydrogels were determined by measuring the percentage of hydrogel mass (%) over time under physiological conditions. CS-CHO(H)/HA-SH had a degradation time approximately twice as long as CS-CHO(L)/HA-SH at 720 min (Figure 3a). This demonstrates that a significant difference in the mechanical properties of this hydrogel system can be achieved simply by increasing the aldehyde functional group content of CS-CHO. As shown in Figure 3b, CS-CHO(H)/HA-SH had a lower but more prolonged swelling property in comparison to CS-CHO(L)/HA-SH due to a higher crosslinking density which resulted in a more compact network structure and thus less water uptake. Even though further detailed studies are required to enhance the mechanical properties of the dynamic hydrogel system to increase the degradation time, both hydrogel systems exhibit good swelling properties. This implies that the hydrogels could protect wounds against the accumulation of excess exudate, implying a probable wound exudate uptake capability.

**Figure 4:**
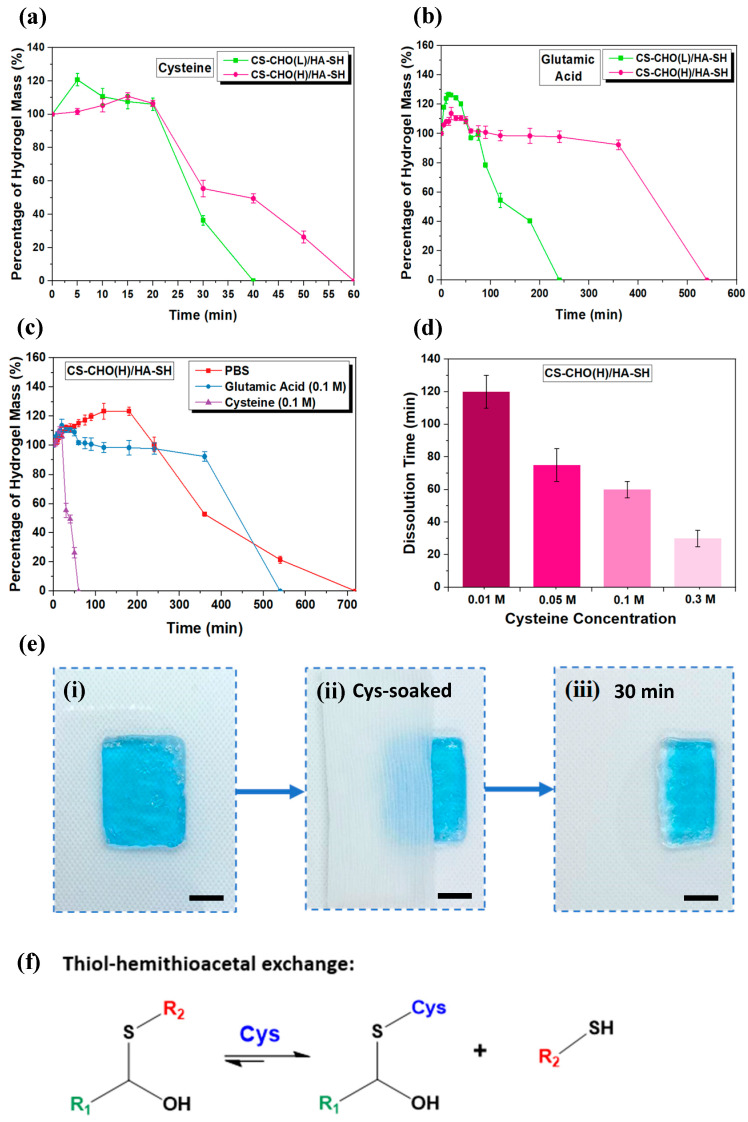
(**a**) Dissolution profiles of CS-CHO(L)/HA-SH and CS-CHO(H)/HA-SH immersed in a cysteine solution (0.1 M, pH 7.4); (**b**) dissolution profiles of CS-CHO(L)/HA-SH and CS-CHO(H)/HA-SH immersed in a glutamic acid solution (0.1 M, pH 7.4); (**c**) dissolution profile of CS-CHO(H)/HA-SH immersed in PBS (pH 7.4), a glutamic acid solution (0.1 M, pH 7.4), and a cysteine solution (0.1 M, pH 7.4); (**d**) dissolution time of CS-CHO(H)/HA-SH hydrogel with different concentrations of cysteine solution (pH 7.4); and (**e**) images of the dissolution of CS-CHO(H)/HA-SH hydrogel after treatment with an aqueous cysteine (Cys) solution (0.3 M, pH 7.4) for 30 min. The hydrogel was stained blue for observation. Scale bar = 1 cm. (**f**) Schematic of the reaction of the dissolution of the hydrogels via a thiol–hemithioacetal exchange reaction.

Mechanical and/or surgical debridement when removing wound dressings can be eliminated by using an on-demand dissolution technique. In the on-demand dissolution study of CS-CHO(L)/HA-SH and CS-CHO(H)/HA-SH, the hydrogels were immersed in PBS (pH 7.4), a cysteine solution (pH 7.4), and a glutamic acid solution (pH 7.4). In the dissolution study using glutamic acid (0.1 M, pH 7.4), a non-thiol-containing amino acid solution was used as the control comparison (Figure 4b). The dissolution profiles in Figure 4a,c show that cysteine (a thiol-containing amino acid solution) could accelerate the dissolution process considerably when compared to the hydrogels immersed in PBS (pH 7.4) only. As expected, the CS-CHO(L)/HA-SH hydrogel degraded faster than CS-CHO(H)/HA-SH due to the lower crosslinking density of CS-CHO(L)/HA-SH. Moreover, the dissolution time was controlled by the concentration of cysteine solution, whereas when the thiol content increased, the dissolution time of CS-CHO(H)/HA-SH could be reduced from ~120 min to ~30 min (Figure 4d). Therefore, by exploiting the thiol–aldehyde addition crosslinks in this hydrogel system, the dissolution mechanism can proceed via thiol–hemithioacetal exchange (Figure 4f) [28]. 

A visual on-demand dissolution study was conducted on the CS-CHO(H)/HA-SH hydrogel two hours after fabrication (Figure 4e(i)). Following this, a cysteine-soaked gauze (0.3 M, pH 7.4) was applied to half of the hydrogel (Figure 4e(ii)). At 15 min, a new cysteine-soaked gauze was applied until complete hydrogel dissolution occurred at approximately 30 min (Figure 4e(iii)). These findings show that the hydrogel’s ability to dissolve on-demand provides a desirable alternative to wound dressing debridement, where a thiol-containing solution would be applied onto the hydrogel. The CS-CHO(H)/HA-SH hydrogels are therefore both injectable for suitable wound attachment and easily removable for changing wound dressings.

### 3.6. In Vitro Biocompatability

To determine the biocompatibility of CS-CHO and HA-SH polymers, HaCaT cells were used for in vitro cell viability tests by an alamarBlue assay. The living status of the HaCaT cells was also confirmed by LIVE/DEAD staining, indicating minimal cytotoxicity in response to the materials. As shown in Figure S3, both CS-CHO and HA-SH showed high cell viability when cocultured with polymer concentrations of 100, 500, and 1000 µg/mL at 24 h and 72 h. However, it is worth noting that CS-CHO concentrations of 500 and 1000 µg/mL showed slightly reduced cell viability when compared to the same HA-SH concentrations. It has been previously reported that the opening of the sugar rings by sodium periodate during the CS-CHO synthesis process can result in reduced cellular recognition of aldehyde-functionalized polysaccharides [33,34].

**Figure 5:**
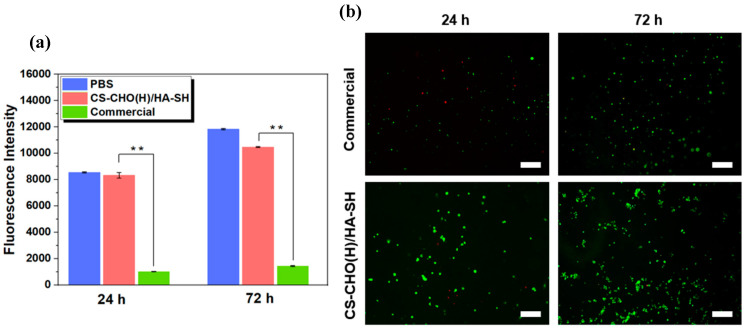
(**a**) Quantitative cell viability evaluation by the alamarBlue assay with NHDFs after being seeded onto CS-CHO(H)/HA-SH hydrogel and a commercial hydrogel (HyStem^®^ Cell Culture Scaffold Kit) for 24 and 72 h; (**b**) representative LIVE/DEAD staining images of NHDFs on the CS-CHO(H)/HA-SH hydrogel and the commercial hydrogel (HyStem^®^ Cell Culture Scaffold Kit) at 24 and 72 h. Live cells are colored green (calcein AM), while dead cells are colored red (ethidium homodimer-1). Scale bar: 50 µm. **, *p* < 0.01.

The biocompatibility of the CS-CHO(H)/HA-SH hydrogel was investigated by seeding Normal Human Dermal Fibroblasts (NHDFs) onto the hydrogel surface and a commercially available HA-based cell culture hydrogel kit (Hystem^®^) at a density of 1.0 × 10^5^/well. Researchers have widely adopted Hystem^®^ hydrogel as a commercially available substrate that can provide a three-dimensional (3D) scaffold for cell culture, therefore it was selected as the control comparison for the viability study [35,36]. The cell viability was evaluated by the alamarBlue assay and imaged by the LIVE/DEAD assay at 24 h and 72 h. Compared to the commercial hydrogel, CS-CHO(H)/HA-SH emitted approximately 5 times more fluorescence at 72 h (Figure 5a), and only a few dead cells (stained in red) were visible in the LIVE/DEAD images (Figure 5b). This result confirms the excellent biocompatibility and proliferation of cells seeded on the CS-CHO(H)/HA-SH hydrogel system, which further suggests the excellent suitability for the hydrogel system to be utilized as a wound dressing.

As demonstrated throughout this study, the dynamic covalent reaction of thiol–aldehyde addition shows great promise for the preparation of dynamic hydrogels. Notably, the dissolution of CS-CHO(L)/HA-SH hydrogels can be significantly accelerated by immersing it in a cysteine solution, which activates the thiol–hemithioacetal exchange mechanism. However, the dissolution rate of a hydrogel immersed in 0.3 M cysteine solution is approximately 30 min, which may be too slow for clinical applications requiring faster dissolution times. Therefore, further studies should investigate the use of higher concentrations of cysteine solution, and other thiol-containing solutions (e.g., glutathione, L-cysteine methyl ester) to achieve faster dissolution. Additionally, adjusting the pH could be explored as a method to optimize the dissolution time. Identifying the optimal conditions for hydrogel dissolution is crucial to ensure the hydrogel’s effectiveness in biomedical applications.

## 4. Conclusions

In this study, a dynamic hydrogel based on thiol–aldehyde addition was designed for wound healing applications using functionalized polysaccharide materials. The dynamic covalent hydrogel offers in situ formation and rapid gelation (within several seconds), resulting in the even coverage of irregular wounds. Furthermore, the thiol–hemithioacetal exchange reaction with a cysteine solution can facilitate the easy removal of the hydrogel dressing within 30 min, avoiding mechanical disruption of the healing process and physical pain for patients. Its dynamic properties and dissolvable nature make it an ideal candidate for wound dressing applications. Furthermore, the hydrogel exhibits excellent biocompatibility and proved to be significant in promoting cell proliferation compared to a commercial hydrogel (HyStem^®^ Cell Culture Scaffold Kit). Therefore, its ability to promote cell proliferation suggests the potential for accelerating wound healing processes. However, given the prevalent challenge of wound infections in clinical settings, future research efforts should prioritize the incorporation of antibacterial properties into these hydrogels to further improve their efficacy in wound care applications. Hence, the CS-CHO/HA-SH hydrogels provide inspiration for the design of dynamic hydrogels for biomedical applications, particularly for wound care treatments as dissolvable wound dressings. 

## Figures and Tables

**Table 1:** Composition of CS-CHO/HA-SH hydrogels.

Hydrogel Sample	CS-CHO (*w*/*v* %)	HA-SH (*w*/*v* %)	Molar Ratio (-SH/-CHO)	Gelation Time (s)
CS-CHO(L)/HA-SH	4.0	4.0	1:0.71	7
CS-CHO(H)/HA-SH	4.0	4.0	1:1.20	5

## Data Availability

Data are contained within the article or the Supplementary Materials.

## Supplementary Materials

The following supporting information can be downloaded at: https://www.mdpi.com/article/10.3390/ma17123003/s1, Supplementary Experimental Section; Figure S1: ^1^H-NMR spectra of HA raw material and HA-SH; Figure S2: Strain-amplitude sweep test of CS-CHO(H)/HA-SH and CS-CHO(L)/HA-SH hydrogels at 2 h; Figure S3: Cell viability evaluation of CS-CHO(H) and HA-SH at different concentrations. Reference [37] is cited in the Supplementary Materials.
