# Supplementary material for: Hyaluronic Acid/Chondroitin Sulfate-Based Dynamic Thiol–Aldehyde Addition Hydrogel: An Injectable, Self-Healing, On-Demand Dissolution Wound Dressing
Source: Materials (Basel). 2024 Jun 19;17(12):3003. doi: 10.3390/ma17123003 (PMC11205580; doi:10.3390/ma17123003)
Supplement: Supplementary file 1 [file materials-17-03003-s001.zip › materials-2880270-supplementary.pdf]

Electronic Supplementary Information (ESI)

Hyaluronic Acid/Chondroitin Sulfate-Based Dynamic Thiol–Aldehyde Addition Hydrogel: An Injectable, Self-Healing, On-Demand Dissolution Wound Dressing

Melissa Johnson [1], Rijian Song [1], Yinghao Li [1], Cameron Milne [1], Jing Lyu [1], Irene Lara-Saez [1], Sigen A [1,2] and Wenxin Wang [1,3,*]

[1] Charles Institute of Dermatology, School of Medicine, University College Dublin, D04 V1W8 Dublin, Ireland

[2] School of Medicine, Anhui University of Science and Technology, Huainan 232001, China

[3] Research and Clinical Translation Center of Gene Medicine and Tissue Engineering, School of Public Health, Anhui University of Science and Technology, Huainan 232001, China

* Correspondence: wenxin.wang@ucd.ie

Table of Contents

Supplementary Experimental Section 3

Synthesis of 3,3'-dithiobis(propanoic hydrazide) (DTP) 3

Figure S1: ^{1}H-NMR Spectra of HA raw material (black line) and HA-SH (red line). 4

Figure S2: Strain-amplitude sweep test of CS-CHO(H)/HA-SH and CS-CHO(L)/HA-SH hydrogels at 2 hours (25 ˚C, and 1 Hz). 5

Figure S3: (a) Quantitative cell viability evaluation by alamarBlue assay of CS-CHO(H) and HA-SH at different concentrations after 24 h co-culture with HaCaTs; (b) Representative LIVE/DEAD staining images of co-culture HaCaTs with CS-CHO(H) and HA-SH (100 µg/mL) at 24 h; (c) Quantitative cell viability evaluation by alamarBlue assay of CS-CHO(H) and HA-SH at different concentrations after 72 h co-culture with HaCaTs; (d) Representative LIVE/DEAD staining images of co-culture HaCaTs with CS-CHO(H) and HA-SH (100 µg/mL) at 72 h. Scale bar: 100 µm. 6

Reference 7

Supplementary Experimental Section

Synthesis of 3,3'-dithiobis(propanoic dihydrazide) (DTP)

DTP was synthesized according to a previously published protocol [1]. Briefly, 3,3'-Dithiodipropionic acid (20 g), absolute ethyl alcohol (200 mL), and two drops of sulfuric acid were added into a round-bottomed flask equipped with a condenser and the system was refluxed overnight until the raw material was fully consumed, monitored by a Thin-layer chromatography (TLC) test. The ethyl alcohol was removed by a rotary evaporator (rotavapor) and diethyl ether (300 mL) was added to dissolve the crude oil. The organic layer was washed with H_2O (3×200 mL), then the diethyl ether was removed by the rotavapor to turn the crude diester (22.8 g) into a colorless oil, and then the diester was used without further purification. The diester (20 g) and hydrazine hydrate (8 equiv.) were dissolved into ethyl alcohol (50 mL). The solution of the diester was added into the solution of hydrazine hydrate dropwise under room temperature (RT). The reaction was heated to 50 °C and monitored by TLC until the reaction was complete, then the solution was cooled to RT. The DTP was precipitated and filtered followed by washing with cold hexane to create the white crystal. The final product was dried in a vacuum oven for 2 days to fully remove the hydrazine hydrate (15.8 g, 88.1% yield). ^{1}H-NMR (400 MHz, DMSO-d6): δ=9.06 (s, 2H), δ=4.22 (s, 4H), δ=2.89 (t, 4H), δ=2.40 (t, 4H).

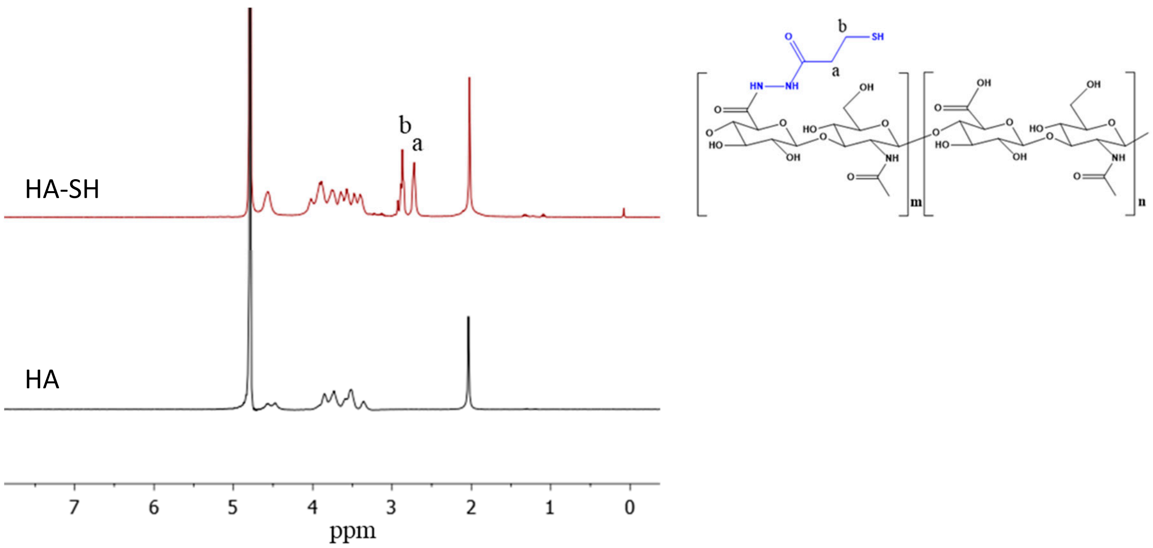

Figure S1: ^{1}H-NMR spectra of HA raw material (black line) and HA-SH (red line).

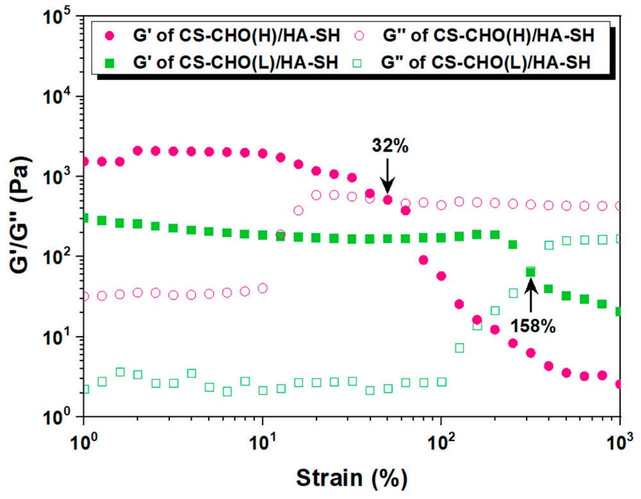

Figure S2: Strain-amplitude sweep test of CS-CHO(H)/HA-SH and CS-CHO(L)/HA-SH hydrogels at 2 hours (25 ˚C, and 1 Hz).

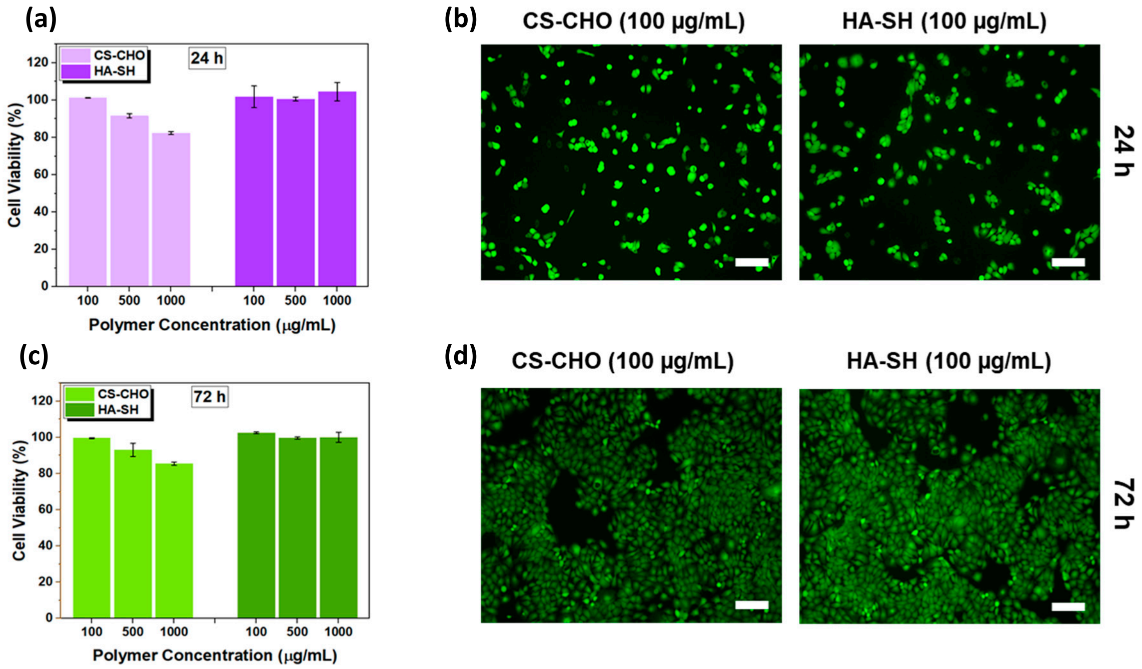

Figure S3: (a) Quantitative cell viability evaluation by alamarBlue assay of CS-CHO(H) and HA-SH at different concentrations after 24 h of co-culture with HaCaTs; (b) representative LIVE/DEAD staining images of co-culture HaCaTs with CS-CHO(H) and HA-SH (100 µg/mL) at 24 h; (c) quantitative cell viability evaluation by alamarBlue assay of CS-CHO(H) and HA-SH at different concentrations after 72 h of co-culture with HaCaTs; and (d) representative LIVE/DEAD staining images of co-culture HaCaTs with CS-CHO(H) and HA-SH (100 µg/mL) at 72 h. Scale bar: 100 µm.

Reference

1 K. P. Vercruysse, D. M. Marecak, J. F. Marecek and G. D. Prestwich, Synthesis and in vitro degradation of new polyvalent hydrazide cross-linked hydrogels of hyaluronic acid, *Bioconjug. Chem.*, 1997, **8**, 686–694.
